# Influence of N-acetyl-L-cysteine against bisphenol a on the maturation of mouse oocytes and embryo development: in vitro study

**DOI:** 10.1186/s40360-019-0323-9

**Published:** 2019-07-22

**Authors:** Qian Li, Zhenjun Zhao

**Affiliations:** College of Life Science, Yantai University, Yantai, China

**Keywords:** Mouse, Oocyte, In vitro maturation, In vitro fertilization, BPA, Oxidative stress, NAC

## Abstract

**Background:**

Bisphenol A (BPA), an endocrine disruptor, is a widely used chemical that has adverse effects on animal development and reproduction. The current research aimed to evaluate the effect of BPA on the in vitro maturation (IVM) and subsequent embryo development of mouse oocytes following in vitro fertilization (IVF).

**Methods:**

IVM was performed in the presence of different concentrations (0, 20, 50, or 100 μg/mL) of BPA. Nuclear maturation, IVF efficiency and embryonic development were determined. The levels of reactive oxygen species (ROS) and glutathione (GSH) in the BPA (50 μg/mL) group were evaluated. We explored the ability of N-acetyl-L-cysteine (NAC) in the IVM medium to rescue the BPA-induced damage by examining changes in nuclear maturation, IVF rate, blastocyst formation, ROS levels and GSH content.

**Results:**

Compared with the control, BPA (50 μg/mL) supplementation during oocyte IVM significantly inhibited nuclear maturation and decreased fertilization and blastocyst formation rates. In addition, BPA exposure increased ROS levels and decreased GSH content in oocytes. The addition of NAC weakened the BPA-induced suppression of nuclear maturation, relieved the BPA-induced downregulation of the fertilization and blastocyst formation rates, and mitigated the increased ROS levels and decreased GSH content.

**Conclusion:**

BPA affects mouse oocyte maturation and subsequent early embryonic developmental competence following IVF by increasing intracytoplasmic oxidative stress in mature oocytes. NAC can reduce these harmful effects to a certain extent.

**Electronic supplementary material:**

The online version of this article (10.1186/s40360-019-0323-9) contains supplementary material, which is available to authorized users.

## Background

Bisphenol A (BPA), an endocrine disruptor [1, 2], is widely used in the production of polycarbonate beverages or food packaging materials and the metal coating on cans. BPA migration into food and beverages could have a negative effect on human health [3]. BPA has been found in some human fluids [4]. BPA exposure causes detrimental effects on development and reproduction by disturbing the oestrous cycle and hormone levels, causing miscarriage, altering oocyte maturation, and compromising oocyte fertilization [4–8]. These adverse effects of BPA exposure have been a cause of concern worldwide.

BPA supplementation inhibits oocyte maturation mostly through disrupting microtubule organization [9–16]. The influence of oxidative stress on the IVM of oocytes has been in discussion, and the results imply that oxidative stress is related to the inhibition of oocyte maturation [17–19]. Liang et al. found that pre-mature ageing of immature oocytes may induce oxidative stress, which inhibits oocyte maturation [20]. The HT-2 toxin induces oxidative stress and inhibits mouse oocyte maturation [21]. Another report showed that BPA exposure could induce oxidative stress, thereby causing DNA damage in INS-1 cells [22]. Few studies have focused on the relationship between the inhibition of oocyte maturation induced by BPA and oxidative stress. One study in which oocytes from mice orally treated with 100 μg/kg body weight (bw)/day BPA and/or 15 or 30 mg/kg bw/day melatonin for 7 days were collected and then cultured for IVM and IVF, it was found that BPA exposure causes oxidative stress and decreases the rate of oocyte maturation and that melatonin protects oocyte maturation [23]. Another study showed that BPA exposure during IVM causes oxidative stress, which is one reason why BPA inhibits porcine oocyte maturation [24]. However, because of differences in the sensitivity of porcine oocytes and mouse oocytes to BPA exposure [24], it is unknown whether BPA addition inhibits the IVM of mouse oocytes by causing oxidative stress.

Furthermore, BPA affects fertility in animal models. Female Sprague–Dawley rats neonatally exposed to 50 μg/50 μL BPA exhibited reduced pup number after mating [25]. Female CD-1 mice treated daily with BPA (0.025 μg/kg bw) perinatally showed reduced fertility and fecundity [26]. Female FVB mice treated with 50 μg/kg bw/day BPA during the perinatal period have smaller litters [27]. In addition, young adult female C57BL/6 J mice exposed to BPA have poorer quality oocytes, leading to reduced fertility following IVF or natural mating [6]. However, whether BPA exposure during mouse oocyte IVM affects subsequent IVF ability and embryonic developmental competence remains unclear.

Reactive oxygen species (ROS) are natural by-products of normal cellular metabolism. Some external environmental factors, such as elevated levels of oxygen tension, increase the cellular production of ROS, which can cause oxidative damage in cells [28, 29]. Excessive ROS can retard oocyte maturation and lead to cell death [30, 31]. Supplementation of the environment with antioxidants could decrease oxidative damage and thus protect cells [32–34]. N-Acetyl-L-cysteine (NAC) is less toxic and can increase intracellular glutathione (GSH) levels [35, 36], and GSH plays a key role in preventing oxidative stress in oocytes [37]. It was reported that oral intake of NAC could increase the intact egg rate of female mice [38]. Compared with the control, 1.5 mM NAC increased the blastocyst rate of porcine zygotes cultured in vitro [39]. Moreover, NAC addition could prevent apoptosis, ageing, and male pronucleus formation of oocytes. NAC (1.5 mM) addition during oocyte maturation also improved the blastocyst rate [40]. However, whether NAC can improve oocyte maturity and development after oocytes exposure to BPA during IVM remains unknown.

Therefore, we investigated the effect of BPA exposure during oocyte IVM on polar body emission, subsequent early embryonic development potential after IVF, ROS levels, and GSH content. Furthermore, NAC was added to the maturation medium to lessen the BPA-induced reduction in meiotic maturation.

## Methods

### Chemicals and culture media

Unless otherwise mentioned, all chemicals used were purchased from Sigma–Aldrich. Dimethyl sulfoxide (DMSO) was used to create BPA stock solutions at different concentrations (20, 50, and 100 μg/mL). The final concentration of DMSO in the culture system was set at 0.1%, and the final BPA dosage used was determined on the basis of our preliminary experiment and a previous report [16]. Ultra-pure water was used to dissolve the NAC stock (200 mM). The final dose of NAC was 100 μM according to our preliminary experiment (see Additional file 1: Figure S1) and a previous report [41]. The ROS Assay Kit was purchased from Beyotime Institute of Biotechnology (Nantong, China), and the GSH detection kit was purchased from Nanjing Jiancheng Bioengineering Institute (Nanjing, China). The maturation medium was tissue culture medium-199 (TCM199; Gibco) supplemented with 10% (v/v) foetal bovine serum (Gibco), 24.2 mg/mL sodium pyruvate, 0.05 IU/mL follicle-stimulating hormone, 1 μg/mL 17β-oestradiol, 0.05 IU/mL luteinizing hormone and 10 ng/mL epidermal growth factor. Human tubal fluid (HTF; Merck Millipore) was used for sperm capacitation. KSOM medium (Merck Millipore) was used for embryo culture.

### Experimental design

To study the effect of BPA on IVM and the subsequent developmental potential of oocytes after IVF, oocytes were treated with various concentrations of BPA (0, 20, 50, and 100 μg/mL) during maturation. After 14 h, the oocytes were subjected to IVF, and embryo development was allowed to proceed (experiment 1). In experiment 2, ROS and GSH levels were determined after oocyte maturation. In experiment 3, the protective effects of NAC on the BPA-induced decreases in oocyte nuclear maturation and embryonic developmental potential following IVF were evaluated. ROS levels and GSH content in oocytes after IVM and treatment with NAC and BPA were also evaluated.

### Animals and oocyte recovery

Female (aged 6–8 weeks) and male (aged 8–10 weeks) Kunming mice (Experimental Animal Centre of Shandong Lvye Pharmaceutical Co., China) were housed in ordinary cages with stainless-steel covers and autoclaved wood bedding. The mice were kept in an air-conditioned room (22 ± 2 °C) with 60 ± 10% humidity under a 12 h/12 h (light/dark) cycle and ad libitum access to food and water. The mice were acclimatized to the housing facility for at least one week before use. At the start of the experiments, female and male mice weighed (mean ± SD) 30 ± 2 and 40 ± 2 g, respectively. 50 mg/mL sodium pentobarbital was diluted 1:10 with sterile water. The mice female and male were anaesthetized by intraperitoneal injection of pentobarbital sodium (50 mg/kg) using a 1-ml plastic syringe. After the mice were asleep, they were killed by cervical dislocation. Fertile male mice were killed to collect spermatozoa from the cauda epididymis for use in IVF. Female mice were killed at 48 h after priming with 10 IU equine chorionic gonadotrophin (Ningbo Hormone Product Co., Ltd., China). The large follicles in the ovary were punctured with M2 medium to release cumulus–oocyte complexes at the germinal vesicle stage. Experimental animals used in this study were cared for in accordance with the standards for laboratory animals. All procedures were approved by the Animal Care of Yantai University.

### In vitro maturation

After being washed three times in M2 and once in TCM199, cumulus oocyte complexes (approximately 30 oocytes per group) were transferred TCM199 drops with different concentrations of BPA (0, 20, 50, or 100 μg/mL) and cultured for 14 h at 37 °C and 5% CO_2_ in humidified air. NAC was added to the IVM medium to observe the effect of antioxidant supplementation. After maturation, cumulus cells were removed from oocytes by pipetting to determine the maturation rate. Oocytes presenting the first polar body were at the metaphase II stage.

### IVF assay and embryo culture

Collected spermatozoa were capacitated in HTF for 1 h at 37 °C. Then, oocytes free of cumulus cells were placed in fertilization medium (30–35 oocytes/100 μL drop) covered with mineral oil. Capacitated spermatozoa (final sperm count 1 × 10^6^/mL) were added to the drop of fertilizing HTF medium. At 6 h after insemination, the oocytes were observed using a stereomicroscope. Oocytes with two pronuclei and two polar bodies were considered fertilized. After insemination, the oocytes were cultured in KSOM medium for 96 h to examine their subsequent development.

### Evaluation of ROS levels

We carried out the ROS analysis according to Nasr-Esfahani et al. [42]. In brief, after being rinsed three times in serum-free TCM199, denuded oocytes were incubated in M2 with 10 μM dichlorodihydrofluorescein diacetate for 10 min at 37 °C. Then, the oocytes were washed and placed on glass slides (approximately 10 oocytes per slide). Fluorescence signals were measured by a laser scanning confocal microscope (emission wavelength of 488 nm), and the fluorescence intensity was analysed using Leica software.

### Evaluation of GSH

Intracellular GSH content was measured as described by Funahashi et al. [43] with modifications. In brief, after being washed with phosphate buffered saline (PBS) three times and rinsed with distilled water three times, denuded oocytes (35 oocytes per group) matured in vitro were evaluated. The freeze-thawed samples were centrifuged (10,000 rpm, 10 min at 4 °C). The supernatant was collected for the GSH assay. The GSH concentration in the oocytes was determined by the 5,5-dithiobis-(2-nitrobenzoic acid)-oxidized GSH reductase recycling assay. The absorbance was measured with a microplate reader at 412 nm. The results are presented as pmol/oocyte.

### Statistical analysis

**Fig. 1:**
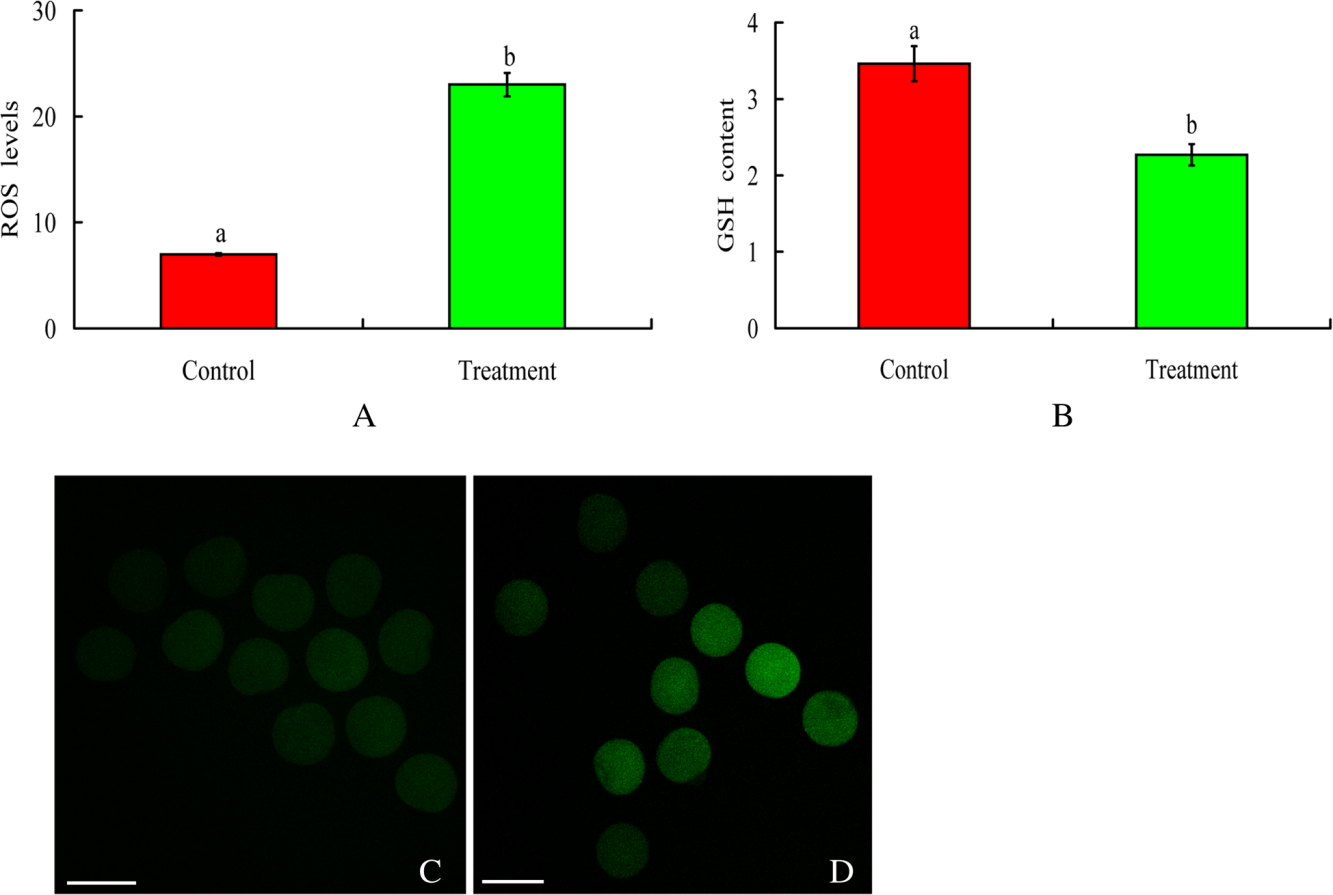
Effect of 50 μg/mL BPA on ROS levels and GSH content in mouse oocytes. **a**. Intracellular ROS levels in mouse oocytes in the control and treatment groups. Oocytes exposed to 50 μg/mL BPA showed higher dichlorodihydrofluorescein fluorescence than those in the control group. ROS levels are presented as the average fluorescence intensity value, which was calculated from the fluorescence intensity values of multiple oocytes. Each treatment was repeated 3–4 times, with each replicate including approximately 10 oocytes. **b**. Intracellular GSH content in mouse oocytes in the control and treatment groups. Oocytes in the 50 μg/mL BPA treatment group had a lower GSH content than those in the control group. Each treatment was repeated 3 times, with each replicate containing approximately 25 oocytes. The data were analysed with an independent-samples T test. ^a, b^Values with different letters above the bars differ significantly (*P* < 0.05). C, control; NAC, 100 μM N-Acetyl-L-cysteine; BPA, 50 μg/mL bisphenol A; NAC + BPA, combined treatment with N-acetyl-L-cysteine (100 μM) and bisphenol A (50 μg/mL). The photographs in C and D show the fluorescence intensity in the control and BPA groups, respectively. Scale bar, 100 μm

Each experiment was repeated at least three times. Data are reported as the mean ± SEM. All data were analysed by SPSS software (SPSS Inc., Chicago, IL). In the BPA group and the control groups (as shown in Fig. 1 a, b), the data of ROS levels and GSH content in oocytes were compared by an independent-samples T test. Other data were analysed by one-way ANOVA with Dunnett’s post-hocpost hoc test. Statistical significance was considered at *P* < 0.05.

## Results

### BPA disturbed oocyte polar body extrusion

The effects of BPA on oocyte polar body extrusion are shown in Table 1. The low dose of BPA at (20 μg/mL) had no effect on the polar body extrusion rate compared to the control. The percentage of oocytes developing to the MII stage was significantly lower with at 50 and 100 μg/mL BPA treatments than with control.

**Table 1:** Effects of BPA on the frequency of first polar body extrusion

Bisphenol A Concentration(μg/mL)	Time (h)	Oocytes (*n*)	Oocytes with polar body extrusion (*n*)	Polar body extrusion rate (% ± SEM)
(Control)0	14	102	96	94.1 ± 1.6^a^
20	14	101	94	93.0 ± 1.1^a^
50	14	103	78	75.7 ± 2.0^b^
100	14	104	41	38.6 ± 3.2^c^

Mouse oocytes were cultured in maturation medium with 0, 20, 50, or 100 μg/mL BPA for 14 h, Then, the number of oocytes with polar body emission was recorded. Each treatment was repeated 3 times, and each replicate contained 30–40 oocytes. The data were analysed with one-way ANOVA and Dunnett’s post- hoc test. ^a-c^Values with different superscripts in the same column are significantly different (*P* < 0.05)

### BPA disrupted the subsequent early embryo development of in vitro matured oocytes

After IVM, the embryo development of oocytes following IVF was studied. The effects of BPA on embryonic developmental competence after IVF are shown in Table 2. No difference in the rates of 4-cell embryos was found among all treated groups. In the BPA (20 μg/mL) group, the rates of fertilization and blastulation were similar to those in the control group. BPA 50 (50 μg/mL) and BPA 100 (100 μg/mL) significantly decreased the fertilization and blastulation rate compared with the control. Based on the results presented in Tables 1 and 2, 50 μg/mL BPA was chosen as the lowest active concentration and used for the following experiments.

**Table 2:** Effect of BPA on subsequent embryo development after IVF

Bisphenol A Concentration(μg/mL)	ooctyes inseminated	oocytes fertilized (%)	4-cell rate (%)	Blastocysts rate (%)
(Control)0	105	86.7 ± 1.0^a^	85.7 ± 1.4^a^	64.8 ± 1.8^a^
20	109	81.1 ± 1.4^a^	84.4 ± 1.4^a^	55.5 ± 1.9^b^
50	107	64.6 ± 2.1^b^	84.7 ± 2.0^a^	44.3 ± 3.0^c^
100	110	38.4 ± 3.0^c^	83.4 ± 1.7^a^	24.1 ± 3.4^d^

After 14 h of maturation, the embryo development of oocytes following IVF was tested. BPA at 50 and 100 μg/mL significantly decreased the rates of fertilization and blastocyst formation. Each treatment was repeated 3 times, and each replicate contained 30–40 oocytes. The data were analysed with one-way ANOVA, followed by Dunnett’s post-hoc test. ^a-d^Values with different superscripts in the same column differ significantly (P < 0.05)

### BPA increased ROS levels in mouse oocytes

After 14 h of IVM, ROS levels in the oocytes were measured. Compared with the control, BPA significantly elevated ROS levels from 7.0 to 23.0 (Fig. 1 a, c, d).

### BPA decreased the intracellular GSH content of mouse oocytes

In the BPA treatment group, the intracellular GSH content was 2.3 ± 0.1, which was lower than that in the control group (3.5 ± 0.2, Fig. 1 b).

### NAC ameliorated the BPA-induced decrease in oocyte nuclear maturation

**Fig. 2:**
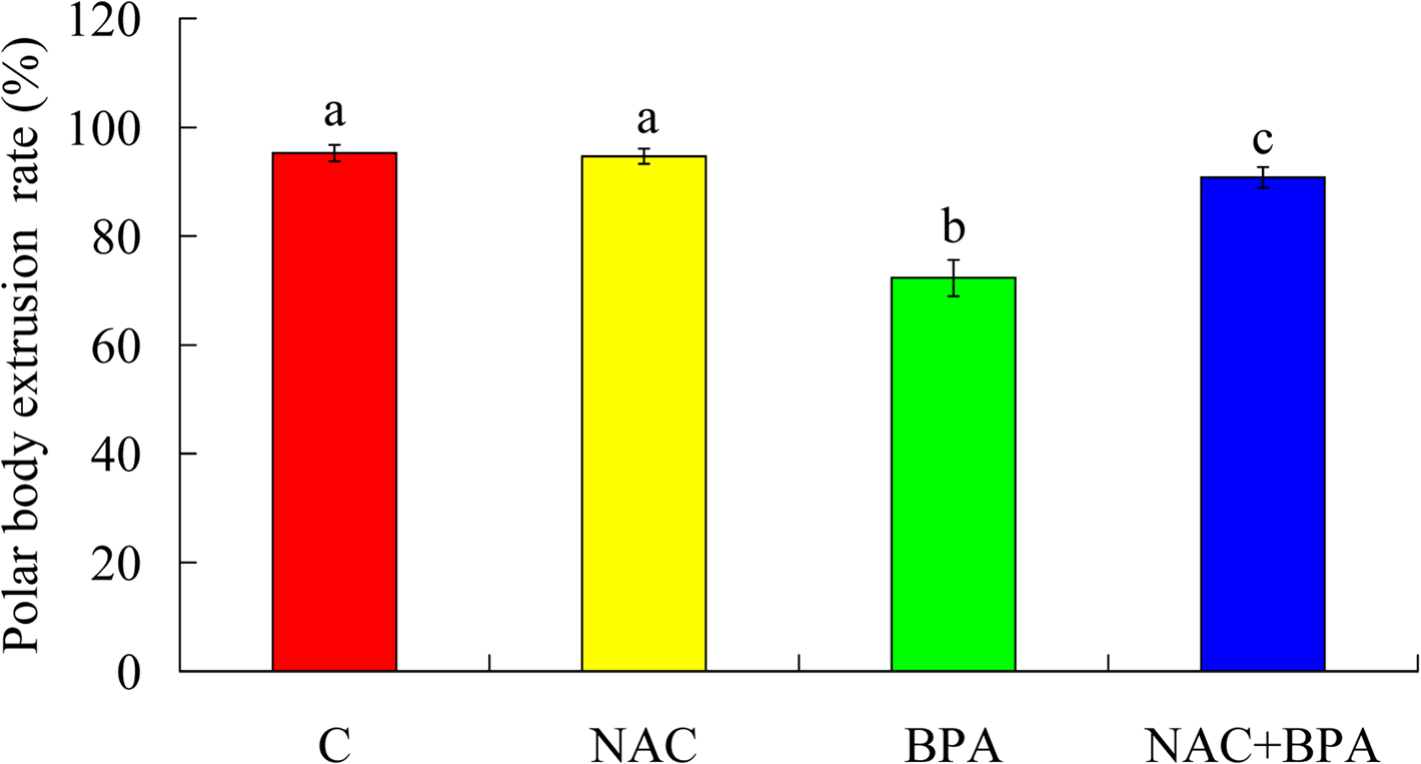
Effect of 100 μM NAC on the inhibition of poly body emission by 50 μg/mL BPA. C, control; NAC, 100 μM N-acetyl-L-cysteine; BPA, 50 μg/mL bisphenol A; NAC + BPA, combined treatment with NAC (100 μM) and BPA (50 μg/mL). The rates of polar body emission (%) in the control, NAC, BPA and NAC + BPA groups were 95.3 ± 1.5, 94.7 ± 1.4, 72.3 ± 3.3 and 90.8 ± 1.9, respectively. After maturation in the presence of 100 μM NAC and,50 μg/mL BPA, the rate of polar body emission in the NAC + BPA co-treated group was somewhat normalized. Each treatment was repeated 3 times, with each replicate containing 30–40 oocytes. ^a-c^Values with different superscripted letters above the bars differ significantly (P < 0.05)

The rate of oocyte nuclear maturation decreased significantly from 95.3 to 72.3% in the BPA-treated group compared with the control group (Fig. 2). The rate of polar body extrusion was 90.8 in the NAC + BPA group, and 72.3 in the BPA group (*P* < 0.05).

### NAC reversed the BPA-induced defect in embryo development after IVF

To investigate the effect of BPA exposure during IVM on the fertilization and developmental competence of the oocytes, we evaluated embryo development following IVF after oocytes matured in medium containing 50 μg/mL BPA alone or in combination with 100 μM NAC. The rates of fertilization and blastocyst formation increased significantly in the NAC + BPA group compared with the BPA group (81.5 vs. 87.0 and 57.0 vs. 45.9, respectively; *P* < 0.05) (Table 3).

**Table 3:** Effect of 100 μM NAC on the 50 μg/mL BPA-induced defects in fertilization ability and embryo development

Groups	oocytes fertilized (%)	Blastocysts rate (%)
Control	87.0 ± 1.1^a^	62.1 ± 3.1^a^
NAC (100 μM)	86.0 ± 1.4^a^	63.1 ± 2.1^a^
BPA(50 μg/mL)	66.0 ± 2.2^b^	45.9 ± 3.0^b^
NAC (100 μM) + BPA(50 μg/mL)	81.5 ± 2.3^a^	57.0 ± 4.1^a^

N-Acetyl-L-cysteine (NAC, 100 μM); Bisphenol A (BPA, 50 μg/mL); NAC + BPA, combined treatment with NAC (100 μM) and BPA (50 μg/mL). After 14 h of maturation in the presence of 100 μM NAC and 50 μg/mL BPA, the oocytes were fertilized in vitro and then cultured to blastocysts. In the NAC + BPA group, the rates of fertilization and blastocyst formation were higher than those in the BPA group. Each treatment was repeated 3 times, and each replicate contained 30–40 oocytes. One-way ANOVA with Dunnett’s post hoc test was used to analyse the data. ^a, b^Values without a common letter above the bars in the same column are significantly different (P < 0.05)

### NAC decreased the BPA-induced increase in ROS levels

**Fig. 3:**
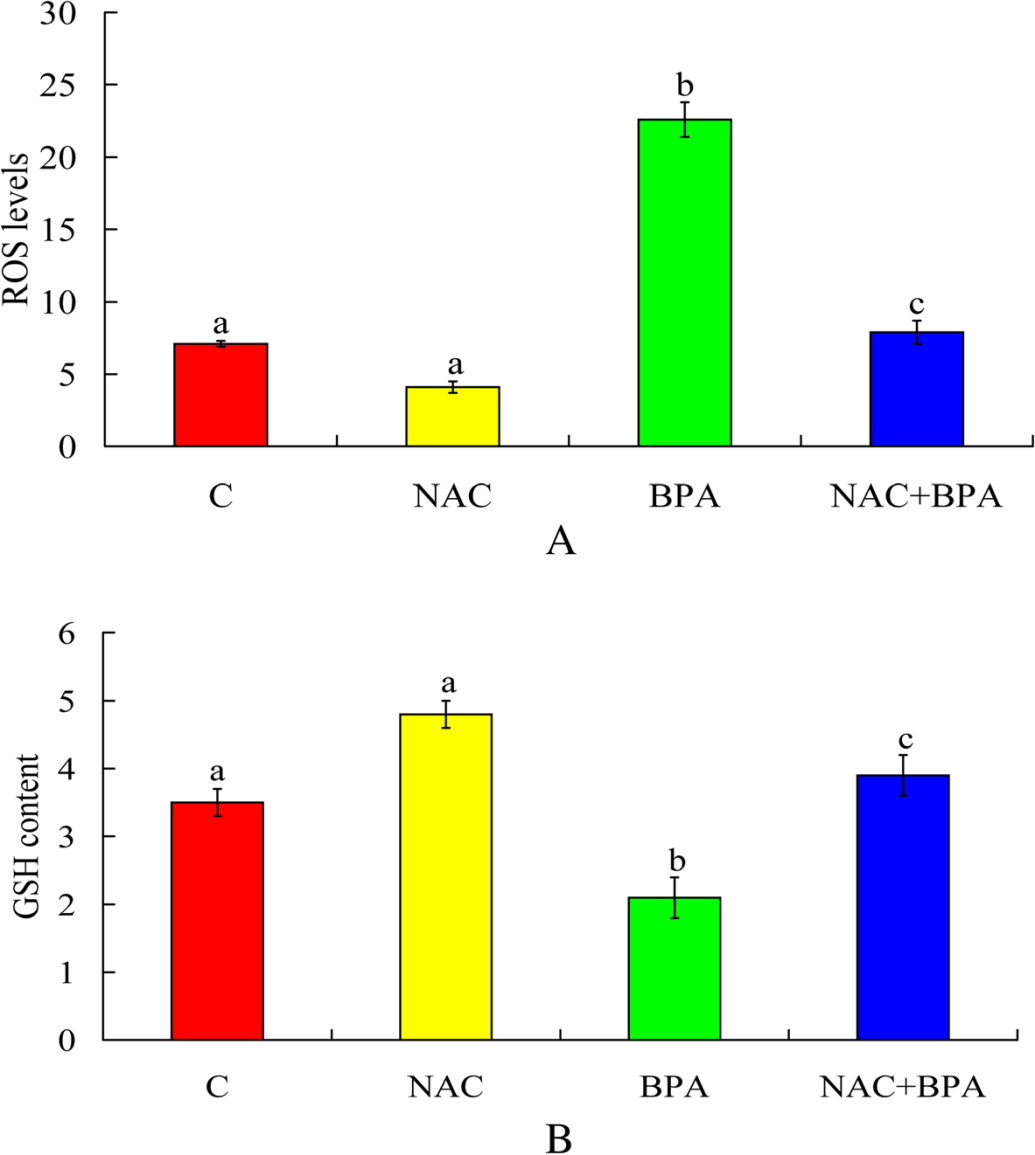
Effect of 100 μM NAC on the ROS increase and GSH decrease induced by 50 μg/mL BPA.C, control; NAC, 100 μM N-acetyl-L-cysteine; BPA, 50 μg/mL bisphenol A; NAC + BPA, combined treatment with N-acetyl-L-cysteine (100 μM) and bisphenol A (50 μg/mL). After 14 h of maturation, (**a**) ROS levels (average fluorescence intensity value) in the control, NAC, BPA and NAC + BPA groups were 7.1 ± 0.2, 4.1 ± 0.4, 22.6 ± 1.2 and 7.9 ± 0.8, respectively. ROS levels significantly decreased in the combination group compared with the BPA group. Each treatment was repeated 3–4 times, with each replicate including approximately 10 oocytes. (**b**) GSH content significantly increased in the combination group compared with the BPA group. Each treatment was repeated 3 times, with each replicate including approximately 25 oocytes.^a-c^Values with a different letter above the bars differ significantly (*P* < 0.05)

After IVM, intracellular ROS levels in mature oocytes were significantly lower in the NAC + BPA group than in the BPA group (Fig. 3 a).

### NAC reversed the BPA-induced decrease in GSH content

The results (Fig. 3 b) showed that the GSH content was significantly lower in the BPA-treated group (2.1 ± 0.3 pmol/oocyte) than that in the control group (3.5 ± 0.2 pmol/oocyte). With NAC and BPA co-treatment, the intracellular GSH content increased to 3.9 pmol/oocyte, compared with the BPA group. The oocytes co-administration with NAC led to a higher intracellular GSH content in oocytes compared with the BPA-treated group alone (NAC + BPA: 3.9 ± 0.3 pmol/oocyte vs. BPA: 2.1 ± 0.3 pmol/oocyte).

## Discussion

The present results showed that BPA inhibited polar body emission, disturbed subsequent fertilization ability and embryo development, increased ROS levels, and decreased intracellular GSH content. NAC could reverse the BPA-mediated inhibition of nuclear maturation and defects in fertilization and embryonic developmental ability. Furthermore, NAC rescued the BPA-induced upregulation of ROS levels and downregulation of intracellular GSH content. To the best of our knowledge, our study is the first to investigate the effect of BPA exposure during IVM on the subsequent embryo development of mouse oocytes following IVF. Moreover, we showed that the reproductive toxicity of BPA can be restored by preventing oxidative stress with NAC supplementation. The study is important in that it identified possible methods to prevent damage induced by BPA exposure during the IVM of mouse oocytes.

Accumulating experimental data have proven that BPA has toxic effects on animal oocyte maturation. Oocyte meiotic progression is an important indicator of high-quality oocytes. In vivo or in vitro BPA exposure causes cell cycle delay in mice [9, 10]. The addition of 50 μM BPA to the culture medium for mouse preantral follicles decreased the maturation rate from 79 to 53% compared with the control [12]. The rate at which porcine oocytes reached the MII stage declined from 81.5 to 53.6% after IVM with 100 μM BPA [13]. BPA (50 and 100 μg/mL) also inhibited the maturation of mouse oocytes cultured for 18 h [16]. Exposure to 20 μM BPA significantly decreased the maturation rate of human oocytes in vitro (19.1%) compared with the control (76.2%) [14]. BPA (30 ng/mL) exposure during bovine IVM decreased the maturation rate from 72.4 to 57.4% compared with the control [15]. These studies suggest that BPA inhibits oocyte maturation, primarily through disrupting microtubule organization. To further clarify the mechanism by which BPA affects oocyte maturation, studies showed that the downregulation of phosphorylated Erk1 (p-Erk1) and phosphorylated CaMKII (p-CaMKII) levels contributes to the decreased oocyte maturation rate [44]. BPA treatment also disrupts porcine oocyte maturation by altering epigenetic modifications and causing oxidative stress and apoptosis/autophagy [24]. A recent study reported that oral administration of BPA (100 μg/kg bw per day for 7 days) decreased the oocyte maturation rate by disrupting spindle organization and chromosome alignment and inducing oxidative stress [25].

Fertilization competence is another important indicator of high-quality oocytes. The current study showed that BPA significantly decreased the rate of IVF. In agreement with a previous study, exposure to BPA (50 μg/kg bw/day) also reduced oocyte fertility after IVF or natural mating in mice [6]. In the current study, BPA addition during oocyte IVM was detrimental to embryo development following IVF. This result agrees with the finding of Ferris et al. [15] that 30 ng/mL BPA supplementation during bovine oocyte IVM could weaken embryonic developmental competence following IVF.

Oxidative stress in oocytes is often determined by measuring ROS levels and GSH content [42, 45, 46]. From the present data, we observed that BPA exposure increased ROS levels and decreased GSH content. These results suggest that BPA exposure results in increased oxidative stress in mouse oocytes. ROS accumulation prevents nuclear maturation and causes cell damage. GSH is an indicator of oocyte cytoplasmic quality. GSH can maintain normal oocyte spindle morphology and help form the male pronucleus. The BPA-induced GSH decrease indicates poor oocyte cytoplasmic quality, which affects subsequent oocyte development. Thus, the decreased developmental potential was possibly caused by oxidative stress resulting from BPA exposure during oocyte maturation. We hypothesized that decreasing oxidative stress during IVM could improve IVF and embryonic developmental competence.

NAC has the ability to facilitate GSH biosynthesis, thus, it can be used as a supplement to replenish GSH during oxidative stress conditions [47]. Mature oocytes need adequate levels of GSH to form a male pronucleus during fertilization [48]. Data indicated that NAC supplementation, specifically during the last 24 h of maturation, in porcine oocyte maturation medium improved male pronucleus formation and blastocyst development [36]. Thus, we propose that NAC could reduce BPA-mediated damage to oocytes. As expected, NAC addition during oocyte IVM largely restored oocyte nuclear maturation and subsequent embryonic development competence following IVF.

Thus, this study showed that BPA impairs mouse oocyte maturation, subsequent fertilization ability and early embryonic developmental competence by increasing intracytoplasmic oxidative stress in mature oocytes, and NAC can mitigate these injuries to a certain extent.

Although this study showed that 100 μM NAC can alleviate BPA-induced damage in mouse oocytes, further study is required to understand the mechanism by which BPA-induced oxidative stress affects nuclear maturation. We hypothesize that the BPA-induced ROS elevation causes oocyte DNA damage and that NAC can alleviate this damage in some way. Moreover, further study is required to evaluate the ability of various antioxidants at different dosages to weaken BPA-induced meiotic arrest, alleviate the disturbance in fertilization and improve the developmental potential of oocytes. In addition, the protective effect of NAC on oxidative stress induced by BPA in mouse oocytes in vivo also needs to be investigated in further studies.

## Conclusion

Our research confirms that BPA negatively affects mouse oocyte maturation, subsequent fertilization ability and early embryonic developmental competence; increases ROS levels; and decreases GSH content. Co-administering NAC and BPA improves mouse oocyte maturation, subsequent fertilization ability and early embryonic developmental competence. Oocytes co-treated with NAC and BPA had lower ROS levels and a higher intracellular GSH content. These results indicate that NAC is suitable for preventing oxidative damage induced by BPA in oocytes.

## Additional file

Additional file 1: **Figure S1.** Effect of NAC on the inhibition of poly body emission by 50 μg/mL BPA. C, control; NAC, 100 μM N-acetyl-L-cysteine; BPA, 50 μg/mL bisphenol A; 50 μM NAC + BPA, combined treatment with N-acetyl-L-cysteine (50 μM) and bisphenol A (50 μg/mL); 100 μM NAC + BPA, combined treatment with N-acetyl-L-cysteine (100 μM) and bisphenol A (50 μg/mL); 200 μM NAC + BPA, combined treatment with N-acetyl-L-cysteine (200 μM) and bisphenol A (50 μg/mL); The rates of polar body emission (%) in the control, NAC, BPA, 50 μM NAC + BPA, 100 μM NAC + BPA and 200 μM NAC + BPA groups were 94.7 ± 1.2, 94.6 ± 2.2, 73.9 ± 2.5, 82.1 ± 2.0, 92.7 ± 1.2, and 91.6 ± 2.9, respectively. Each treatment was repeated 3 times with each replicate containing 30–40 oocytes. ^a-c^Values with different letters in their superscripts above the bars differ significantly (*P* < 0.05). (DOC 1317 kb)

## Data Availability

The datasets used and analysed during the current study are available from the corresponding author upon request.

## Abbreviations

BPA: Bisphenol A
DMSO: Dimethyl sulfoxide
GSH: Glutathione
IVF: In vitro fertilization
IVM: In vitro maturation
NAC: N-Acetyl-L-cysteine
PBS: Phosphate buffered saline
p-CaMKII: Phosphorylated CaMKII
p-Erk1: Phosphorylated Erk1
ROS: Reactive oxygen species
