# Supplementary material for: Influence of N-acetyl-L-cysteine against bisphenol a on the maturation of mouse oocytes and embryo development: in vitro study
Source: BMC Pharmacol Toxicol. 2019 Jul 22;20:43. doi: 10.1186/s40360-019-0323-9 (PMC6647297; doi:10.1186/s40360-019-0323-9)
Supplement: Supplementary file 1 — Figure S1. Effect of NAC on the inhibition of poly body emission by 50 μg/mL BPA. C, control; NAC, 100 μM N-acetyl-L-cysteine; BPA, 50 μg/mL bisphenol A; 50 μM NAC + BPA, combined treatment with N-acetyl-L-cysteine (50 μM) and bisphenol A (50 μg/mL); 100 μM NAC + BPA, combined treatment with N-acetyl-L-cysteine (100 μM) and bisphenol A (50 μg/mL); 200 μM NAC + BPA, combined treatment with N-acetyl-L-cysteine (200 μM) and bisphenol A (50 μg/mL); The rates of polar body emission (%) in the control, NAC, BPA, 50 μM NAC + BPA, 100 μM NAC + BPA and 200 μM NAC + BPA groups were 94.7 ± 1.2, 94.6 ± 2.2, 73.9 ± 2.5, 82.1 ± 2.0, 92.7 ± 1.2, and 91.6 ± 2.9, respectively. Each treatment was repeated 3 times with each replicate containing 30–40 oocytes. a-cValues with different letters in their superscripts above the bars differ significantly (P < 0.05). (DOC 1317 kb) [file 40360_2019_323_MOESM1_ESM.doc]

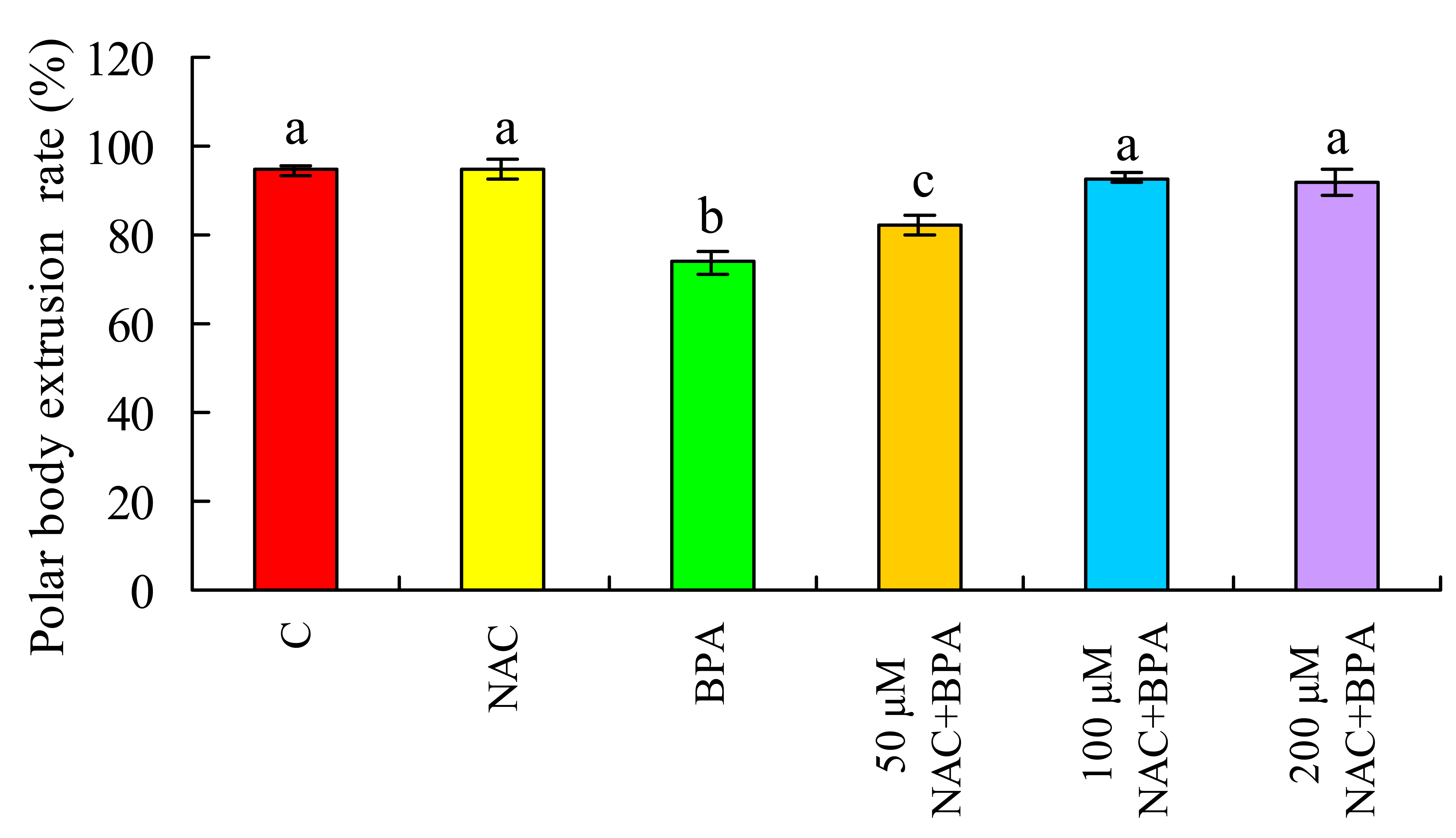


**Figure.**  Effect of NAC on the inhibition of poly body emission by 50 μg/mL BPA. C, control; NAC, 100 μM N-acetyl-L-cysteine; BPA, 50 μg/mL bisphenol A; 50 μM NAC+BPA, combined treatment with N-acetyl-L-cysteine (50  μM) and bisphenol A (50 μg/mL); 100 μM NAC+BPA, combined treatment with N-acetyl-L-cysteine (100  μM) and bisphenol A (50 μg/mL); 200 μM NAC+BPA, combined treatment with N-acetyl-L-cysteine (200  μM) and bisphenol A (50 μg/mL); The rates of polar body emission (%) in the control, NAC, BPA, 50 μM NAC+BPA, 100 μM NAC+BPA and 200 μM NAC+BPA groups were 94.7±1.2, 94.6±2.2, 73.9±2.5, 82.1±2.0, 92.7±1.2, and 91.6±2.9, respectively. Each treatment was repeated 3 times with each replicate containing 30-40 oocytes. ^a-c^Values with different letters in their superscripts above the bars differ significantly (P < 0.05).
